# Modeling Uncertainties in EEG Microstates: Analysis of Real and Imagined Motor Movements Using Probabilistic Clustering-Driven Training of Probabilistic Neural Networks

**DOI:** 10.3389/fnhum.2017.00534

**Published:** 2017-11-01

**Authors:** Martin Dinov, Robert Leech

**Affiliations:** Computational, Cognitive and Clinical Neuroimaging Laboratory, Division of Brain Sciences, The Centre for Restorative Neuroscience, Imperial College London, London, United Kingdom

**Keywords:** EEG, probabilistic microstates, K-Means, Fuzzy C-Means, multi-layer perceptrons, motor imagery

## Abstract

Part of the process of EEG microstate estimation involves clustering EEG channel data at the global field power (GFP) maxima, very commonly using a modified K-means approach. Clustering has also been done deterministically, despite there being uncertainties in multiple stages of the microstate analysis, including the GFP peak definition, the clustering itself and in the post-clustering assignment of microstates back onto the EEG timecourse of interest. We perform a fully probabilistic microstate clustering and labeling, to account for these sources of uncertainty using the closest probabilistic analog to KM called Fuzzy C-means (FCM). We train softmax multi-layer perceptrons (MLPs) using the KM and FCM-inferred cluster assignments as target labels, to then allow for probabilistic labeling of the full EEG data instead of the usual correlation-based deterministic microstate label assignment typically used. We assess the merits of the probabilistic analysis vs. the deterministic approaches in EEG data recorded while participants perform real or imagined motor movements from a publicly available data set of 109 subjects. Though FCM group template maps that are almost topographically identical to KM were found, there is considerable uncertainty in the subsequent assignment of microstate labels. In general, imagined motor movements are less predictable on a time point-by-time point basis, possibly reflecting the more exploratory nature of the brain state during imagined, compared to during real motor movements. We find that some relationships may be more evident using FCM than using KM and propose that future microstate analysis should preferably be performed probabilistically rather than deterministically, especially in situations such as with brain computer interfaces, where both training and applying models of microstates need to account for uncertainty. Probabilistic neural network-driven microstate assignment has a number of advantages that we have discussed, which are likely to be further developed and exploited in future studies. In conclusion, probabilistic clustering and a probabilistic neural network-driven approach to microstate analysis is likely to better model and reveal details and the variability hidden in current deterministic and binarized microstate assignment and analyses.

## Introduction

**Figure 1:**
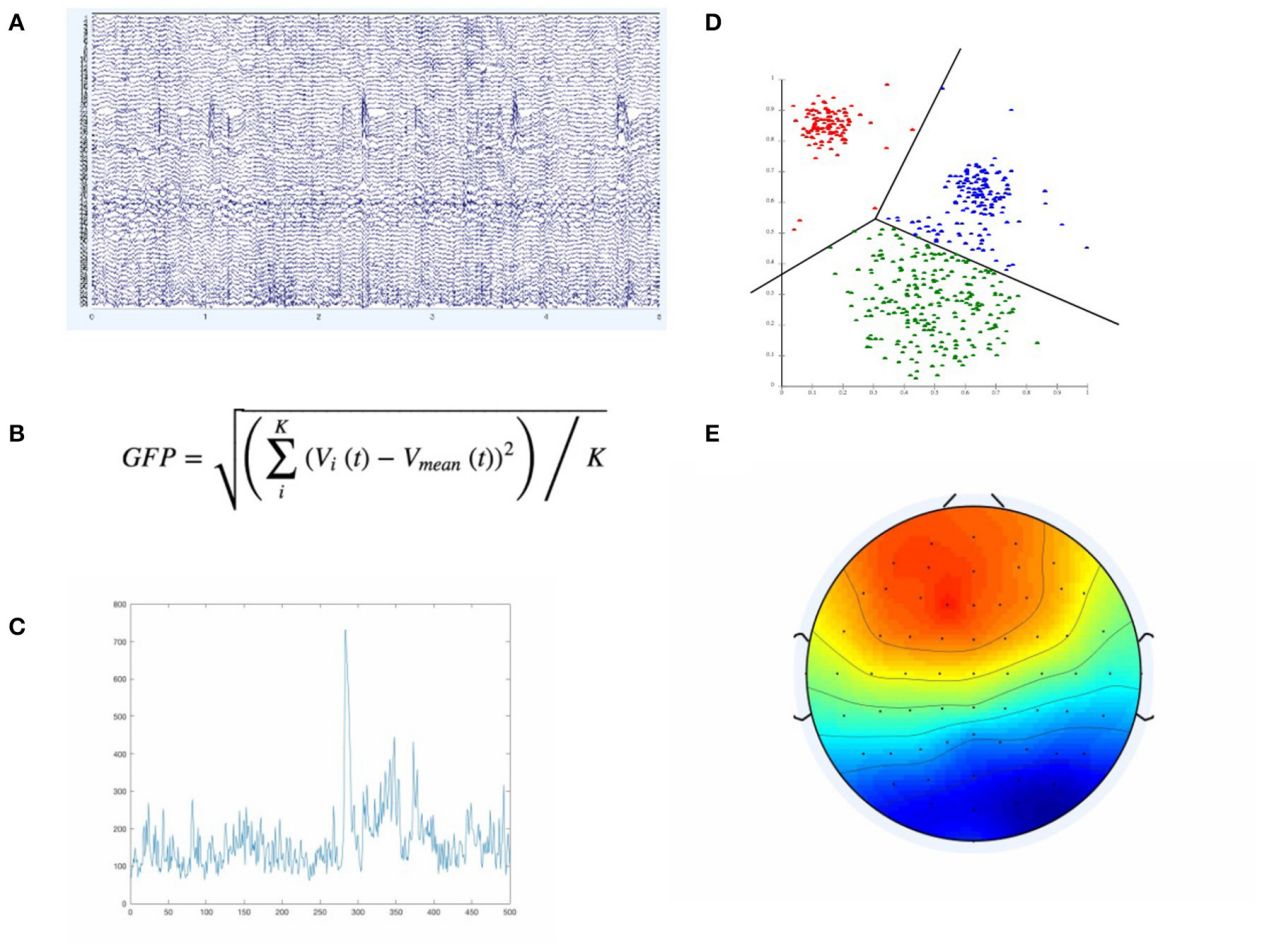
The Figure illustrates the main elements involved in defining microstate template maps E. We start with the actual EEG data **(A)**, compute, at each time point, the GFP according to the equation in **(B)**. If we plot the resulting GFP curve, we would only have a single GFP curve as in **(C)**. If we then extract the EEG samples only at GFP peaks (GFP maxima), we feed those samples (of higher SNR than neighboring regions) into a clustering algorithm—typically K-Means, depicted in **(D)**. The resulting microstates (i.e., clusters) have a characteristic spatial distribution across the EEG sensors, as in **(E)**.

Electroencephalography (EEG) is one of the most widely used and practical brain imaging modalities. While there are well-recognized and fundamental limitations to EEG spatial resolution, it is the easiest to access, cheapest and most portable of the two main high temporal resolution brain activity-recording technologies (the other being magnetoencephalography or MEG), ensuring that EEG has remained the most viable approach to monitoring brain activity “in the field” (Casson et al., 2010; Burle et al., 2015). While many approaches exist for analyzing EEG, for example, in Brain Computer Interfaces (BCIs), over the last decade the multivariate approach of microstate analysis has surged in popularity; providing a relatively simple but expressive way of providing a functional description of the global brain state and neural dynamics (Lehmann et al., 1987; Khanna et al., 2015). In the simplest and most common case, microstate analysis proceeds as shown in Figure 1 below:

First, the global field power (GFP) is computed over the time series of interest. The GFP represents the spatial standard deviation across all channels and is a reference-free measure, in that regardless of how the channels are referenced, leads to the same result (Khanna et al., 2015). This makes it useful in determining certain global and reference-invariant properties from the EEG. In particular, maximum points on the computed GFP curve should constitute points of maximal signal-to-noise ratio (Lehmann et al., 1987; Khanna et al., 2015). By using the EEG samples only at those GFP maxima, we maximize the signal-to-noise ratio of the template maps that we are to compute from the EEG. The GFP-maxima-derived EEG samples are then typically fed into a clustering algorithm, most commonly a modified K-Means (KM) algorithm, which ignores polarity, first proposed and developed in (Pascqual-Marqui et al., 1995). The resulting cluster centroids computed by this modified-KM algorithm represent template maps that can be assigned, according to topological similarity, back onto each of the EEG samples/time points of the full EEG time series. These microstate template maps are typically computed over the whole group and across conditions, thus representing group template maps. There are different approaches to categorizing/assigning each point in the EEG time series a microstate label, but the most common approach is some type of spatial correlation between the group-level template maps and the subject-level EEG time points (Van De Ville et al., 2010; Drissi et al., 2016). This assignment of label to the current EEG time point by using the maximally correlated of all the template maps is deterministic and equivalent to selecting the template that matches best according to the maximum likelihood of the template map-derivation model used. Since the original proposal of this KM-based approach in (Pascqual-Marqui et al., 1995), other researchers have also used other ways in deriving template maps/microstate maps that differ from the standard K-means (KM) approach—most notably ICA (Musso et al., 2010; Yuan et al., 2012) and agglomerative hierarchical clustering (Britz et al., 2010; Van De Ville et al., 2010). However, all approaches currently used are fundamentally throwing away information and over-simplifying the underlying dynamics and models by being deterministic. While microstates assignment has been defined as “all-or-none” and belonging to a given microstate scalp topography template is considered binary, this has certainly missed on some of the uncertainty in the underlying dynamics and the assignment of a given template to a given timepoint in the EEG timecourse.

Over the interval of ~100 ms, the measurable scalp topography is reported to be relatively stable, and so the brain is considered to be in a quasi-stable microstate (Pascqual-Marqui et al., 1995; Khanna et al., 2015). Therefore, any assigned state label is assumed to be unchanging for approximately 80–120 ms. After a period of time on the order of this duration, the scalp electrical topography rapidly (but not randomly—but rather probabilistically) shifts to one of the other configurations or microstates for roughly the same amount of time. Furthermore, as the EEG signal involves large amounts of oscillatory activity, only the actual scalp topography of microstates is considered, regardless of the polarity (i.e., flipping polarity of a map is considered to still be part of the same microstate, as the relative topography is unchanged, when the polarity of the signal is ignored). This is a large part of the motivation behind the modification of the standard K-means algorithm as developed in and proposed by (Pascqual-Marqui et al., 1995). One of the issues of using a standard K-Means algorithm is that EEG points with highly similar scalp topography but with inverted polarity are in general going to be classified as belonging to different map clusters. The dynamics of microstates (such as the exact microstate label, the duration, or the specific transition frequencies/probabilities between microstates) change between cognitive conditions, tasks, age, sex, and pathologies (Britz et al., 2010; Lehmann et al., 2010; Musso et al., 2010; Khanna et al., 2015; Drissi et al., 2016; Milz et al., 2016). They have also been found to have scale-free dynamics and strong relationship to various blood oxygen level dependent (BOLD) fMRI (functional magnetic resonance imaging) networks (Musso et al., 2010; Van De Ville et al., 2010). Microstates seem to predict the activity of certain resting state networks (RSNs) and task networks, despite the slower temporal scales of BOLD imaging. We have previously (Dinov et al., 2016) found some weak suggestive evidence that microstate dynamics may be predictive of and predicted by EEG avalanche/cascade dynamics. The latter have been shown to exhibit scale-free behavior across orders of magnitude, both in simultaneous EEG-fMRI (Fagerholm et al., 2015) and in separate EEG (Benayoun et al., 2010; Palva et al., 2012) studies. As such, microstates relate to a number of other measures of brain dynamics in theoretically, cognitively and clinically useful ways. Microstates are therefore an exciting multi-faceted area of neuroimaging, experimental, clinical and cognitive neuroscience research, which we are now exploring.

Although microstates are defined using data-driven clustering, they typically result in a consistent set of topographic maps that are “canonical” or commonly reported in the literature; **Figure 4B** shows 4 such canonical microstates, commonly found reported in the relevant literature. However, there are nevertheless many issues with most clustering approaches that will be briefly mentioned. While we focus here on K-Means, many of these remarks are valid for other clustering (and non-clustering) techniques too. Many of the algorithms employed for deriving template maps are susceptible to local optima, physiological relevance (or lack thereof) of the number and type of clusters chosen, the amount of data required to obtain good results, the many (sometimes strong) assumptions the algorithm makes, and others. As such, there are a number of algorithmic and data-related sources of noise that are currently not taken into account, which could be informative in further delineating brain microstate properties. As such, we highlight three (of probably more) issues here that are problematic with the current approach to microstate studies:

- GFP peak detection will detect very small “hills” (that barely differ from neighboring GFP points) as GFP maxima (as they technically are maxima compared to the surrounding GFP points). However, such GFP peaks will only represent a small (possibly negligible) difference in SNR. Such points should not be included straightforwardly into whatever clustering or grouping method is used during template map building. They could be weighted differentially, for instance. This may reduce the certainty in any given label assignment, depending on how close or how far we are from such GFP “small maxima.” There may be other GFP-related effects on the microstate predictability.
- If KM or similar clustering algorithms are not run with sufficiently many replications or for sufficiently many iterations, the solutions found will not be optimal for the chosen number of clusters and the data set in question. This would should then increase uncertainty in the downstream analysis steps.
- Even if the global minimum of the error function used in the algorithm is found, there may be overlap and more or less of similarity between the clusters and the “optimum” solution may not be mathematically, biologically and physiologically realistic if we assume that a given time point belongs strictly to one cluster. Overlap between clusters would be indicative that the notion of “the closest/most similar cluster” may be misleading and removing informative detail about the underlying dynamics or predictability/certainty of the microstates. It is well known that KM is notoriously bad at finding “correct” clusters in many data distributions.

In this paper, we used a standard (non-modified) K-Means algorithm and ensured post-clustering that the final maps were not duplicate due to polarity inversion. We then applied an alternative probabilistic clustering approach, an analog to K-means, called fuzzy C-means (FCM), which naturally extends KM to incorporate probabilistic cluster label assignments to each point, giving the degree of membership to each of the found clusters/templates for each point. We did not use the modified K-Means of (Lehmann et al., 1987) but instead used the standard MATLAB K-Means implementation and eliminated one template map found that was an inversion of one of the other templates. Standard KM was used to allow for an easier and more direct comparison to FCM, as the standard KM is almost identical to FCM except for two terms. While FCM will suffer from many of the same issues as KM, it does not assume deterministic and absolute membership to any given cluster and solves one of the major issues of KM. Probabilistic cluster assignments therefore allows us to quantify how certain or uncertain we are in any given microstate label assignment and subsequent analysis of microstates relying on those microstate labels. As such, this is an improvement on standard deterministic approaches and it provides a good probabilistic method to compare against KM, the latter being one of the most commonly employed clustering methods in the field.

We trained Multi-Layer Perceptrons (MLPs) with the data to be clustered serving as inputs and the cluster labels serving as the target values to use for the supervised training. The rationale for using MLPs was to probabilistically assign a template/cluster to each EEG time point from the real and imagined motor movement data. This now allows us to very quickly (e.g., in real-time settings) and robustly assign probabilistic microstate labels to the EEG time series. A neural network-driven microstate assignment approach also allowed us to quantify in a novel and straightforward way the relationship between the mean GFP of a subject and the predictability of the current microstate assignment as assigned by the MLPs.

While EEG has had some limited use outside of clinical and research contexts, it has typically relied on standard EEG analysis, usually involving some kind of classification on specific types of input data (e.g., classification on motor imagery) or, even more commonly and historically, some fourier/band amplitude-based measure of relaxation. While these have shown some usefulness for very simple applications, there is only very limited use of EEG technology outside of clinical, research and experimental settings or for more complex requirements. Though the technology is now relatively widespread, easy to use and affordable, one reason for the lack of broader application in real-world settings may be the lack of an easily and readily interpretable, or meaningful, and easily derivable measure from the EEG. We were interested in investigating approaches to improving EEG microstate usefulness for portable EEG applications, as microstates are beginning to be better understood, are easily computable and potentially useable in real-time. In particular, the development and use of a fully probabilistic framework for microstate assignment and analysis, as we propose here, should allow for the variability or uncertainty in the current microstate assignment to be taken into account in future models of microstate dynamics.

## Methods

### Overview and data used

We used motor imagery data from PhysioNet, contributed by the Schalk lab (Goldberger et al., 2000; Schalk et al., 2004) that is available for public use. It is fully anonymized data available to all researchers for free use under the ODC Public Domain Dedication and License v1.0. It consists of 109 subjects doing a motor movement and imagery task. We have used a large subset of their entire data set, consisting of the imagined and the real motor movements of both hands or feet—i.e., for the task, they either squeezed both of their hands or both of their feed, signified by visual signals on a screen in front of them. We ignored the eyes open, eyes closed, as well as left and right imagined and real motor movements, leaving us with a total of the 109 subjects with 3 replicate sets of imagined and real motor movements of both hands or both feet (alternating within multiple task blocks for each subject), i.e., 327 sets of EEG recordings for real motor movements and 327 sets of EEG records for imagined motor movements. The data was recorded with a 64-channel BCI2000 system according to the 10-10 in EDF+ format. There were no electrooculogram (EOG) channels, so we used an automatic EOG artifact removal method based on ICA, as described in (Gomez-Herrero et al., 2006) for the cleaned-data version of the analysis. We report in part both pre-cleaning and post-cleaning results, as there are complementary results from both—though the results from the artifact-cleaned data are reported more fully. We supplied no manually-chosen parameters to the EOG-cleaning method, letting it automatically derive the assumed-optimal parameters in a fully data-driven way. The approach is implemented as the Automatic Artifact Removal (AAR) plugin in the EEGLAB toolbox, which we used for importing and using the EDF+ EEG data files. We also manually verified for the first three subjects' data sets that the approach was working well and comparable to a manual ICA-based removal of EOG components, i.e. the AAR approach and manually removing EOG components from a manual ICA results in very similar maps and cleaned EEG traces. Furthermore, the AAR-cleaned data removed a number of ocular artifact template maps that were previously present in the data, leaving us with maximally non-artefactual maps for the cleaned data. The maps are show in in **Figure 5** for pre-cleaned template maps and 6 showing the resulting 4 maps post-EOG and EMG cleaning.

**Figure 2:**
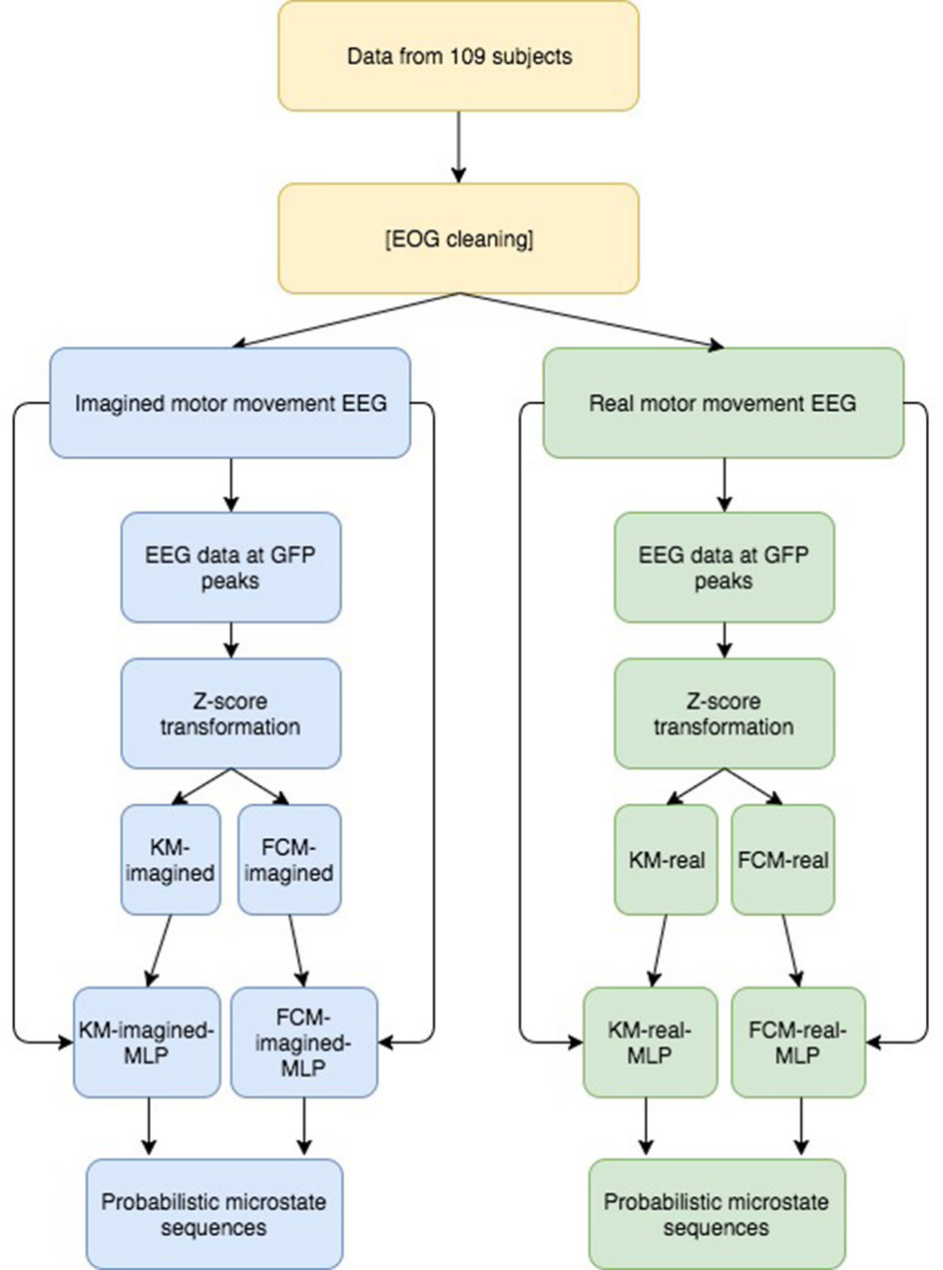
Overview of our approach to derive probabilistic microstate sequences. Briefly, we performed the same analysis on both imagined and real motor imagery data and we report results for both uncleaned and cleaned data, showing a similar story. After GFP computation and subsequent peak detection, we Z-transformed the EEG data at those peaks, fed those Z-transformed data points into 2 clustering algorithms (KM and FCM), then, using the resulting labeled data points, we trained 2 MLPs for both imagined and real motor imagery data (for a total of 4 MLPs), which were then used to derive probabilistic microstate sequences for all 4 cases. We then look at similarities and differences between the final probabilistic microstate sequences.

For both imagined and real motor movements, the subjects had their eyes open and were looking at a screen, which instructs them whether to open or close (or imagine doing so—for the imagined movement cases) both of their hands into fists or curl up both of their feet. We excluded a few of the data sets due to missing, broken and noisy data, leaving us with 320 data sets for real motor movements and 325 for imagined motor movements. Given the high number of points (>1.5 million for both imagined and real) the ratio of real to imagined data points are equal enough (~1:1) to not bias model training toward either imagined or real motor movement data. We then computed the GFP and fed the zscore-normalized EEG samples at the GFP maxima into FCM and KM. One of each model (FCM and KM) was used for imagined motor movement data and for real motor movement data for a total of 4 models (FCM-imagined, FCM-real, for imagined and real motor movements, respectively, and similarly for KM with KM-imagined, KM-real). Figure 2 visualizes the overall analysis just described here.

### K-means clustering as the standard to compare against

**Figure 3:**
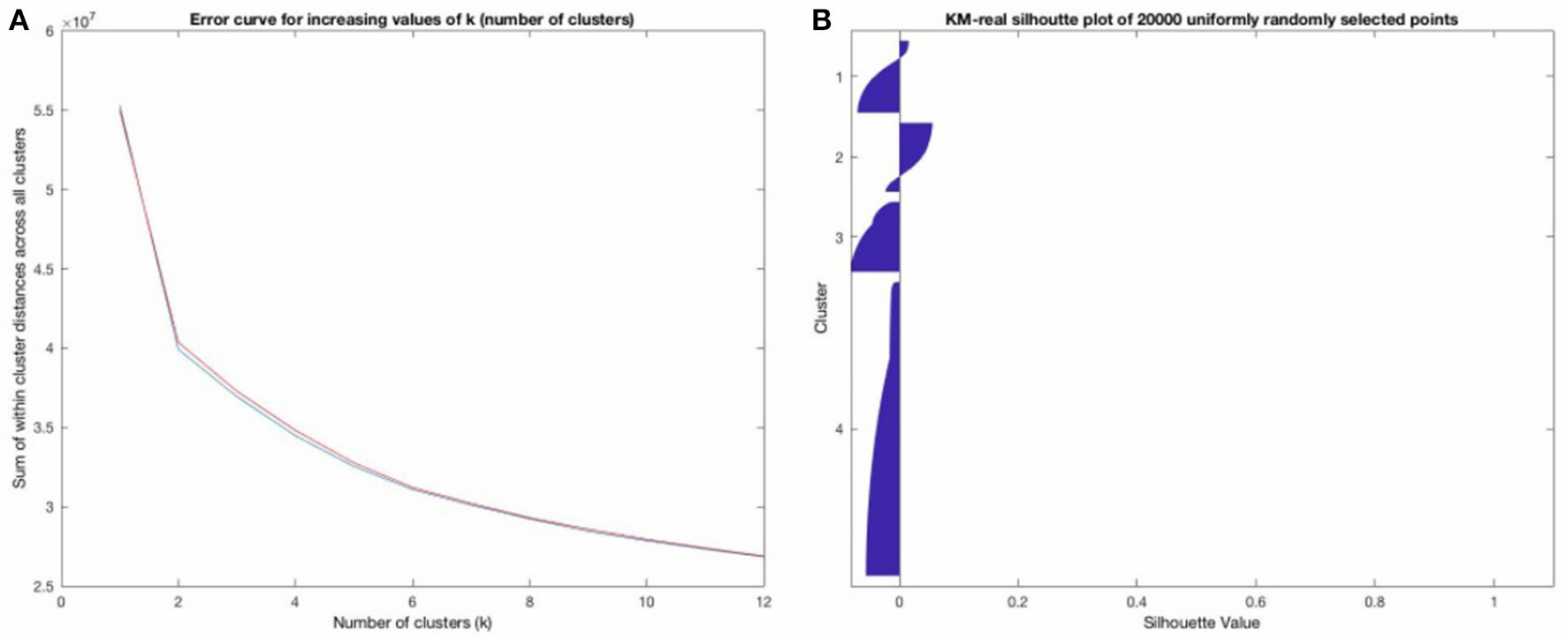
**(A)** shows the error plot as a function of increasing k (number of clusters). **(B)** above shows a “Silhouette plot” that suggests that though the clustering was “optimal,” it is, in fact, moderately uncertain, with most points not having a high Silhouette value at all (see the description of how this is computed). **(B)** strongly suggests that a probabilistic clustering and assignment approach should be used, taking these inherent uncertainties into account.

We first ran standard K-Means (kmeans++) with a pre-determined number of clusters (*k* = 9) for a maximum of 5,000 iterations (or until convergence of MSE (of the Euclidean distance) of all the points to their respective clusters) with 10 replicates on the raw EEG data. We present both raw/uncleaned and cleaned results, in part to show that most of the template maps found post-cleaning are also found in the uncleaned EEG data. This is useful in that if neural networks can be developed to assign template maps on uncleaned EEG data, this would potentially allow for an even more real-time use of microstates. The model (the 9 64-dimensional vectors representing the template maps or centroids determined by the algorithm) is chosen according to the set of clusters generated with the lowest error from all the replicate runs. This strongly increases the probability that the found models represent the global maximum (as opposed to a local maximum) of the error function and that they are therefore the “best” template maps that KM with the given number of clusters can find. We repeated this a few times to ensure that there is consistency in the results used. We further noted that the lowest objective function error was achieved on at least 3 of the 10 replicates for KM-real (3 for the final run of KM-real used for the results), increasing the likelihood that the repeating minimum of error found (on 3 of the runs) is in fact the global minimum of error and therefore the optimal clustering, given this data and this objective function—i.e., this is the best clustering solution that KM can provide for this data given this number of clusters chosen. Originally, *k* = 4 number of template maps has been used, but in more recent and “identified” 4 more or less meaningful template maps using his modified K-Means algorithm. Since then *k* = 4 has been used, but in more recent years, by comparing EEG and fMRI, we've “identified” over 20 more or less meaningful maps. Some of these are “new” and others are likely a combination or sub-maps of the original 4 that Lehmann found. As with other unsupervised approaches like ICA, using a very high number of clusters or components is likely to split a single well-behaved (i.e., low within-group variance) cluster into multiple otherwise related components. Also, maximizing for explained variance on a given specific data set leads to over-fitting to that data set (and decreasing generalization of the involved models). We plotted the total distance of the points within a cluster (the sum of the within cluster distances to their assigned cluster, summed across all the clusters and all the points) for increasing values of k (= the number of clusters used), using 10 replicates for each cluster size. We did this for KM-real and KM-imagined and let the resulting decision drive the decision for the number of clusters to use in FCM, so as to make FCM's results maximally comparable to KM's. This is shown in Figure 3A for the EOG-cleaned data. We also plotted Silhouette plots (Kaufman and Rousseeuw, 1990), which show the “goodness of belonging” of any point to the given cluster, across a subset of the points for KM-real and KM-imagined, shown in Figure 3B (again for the cleaned-version of the data). The silhouette value for a point i is computed according to the Equation:

$$\begin{array}{l} S_{i\;}=\frac{\left(b_{i}-a_{i}\right)}{\max\left(a_{i},\;b_{i}\right)} \end{array}$$

where *b*_*i*_ is the minimum mean distance from the i-th point to the points in the different clusters, minimized over all clusters. *a*_*i*_ is the mean distance from the i-th point to the other points in the same cluster as this point. In other words, the silhouette value gives a single value, for each point in the points plotted on a silhouette graph, which gives us a measure of how well that point was matched to a given cluster, according to all the other points in that cluster and according to the points in the other clusters. As *S*_*i*_ ∈ [−1, 1], a value toward 1 indicates a very good match of that point to its assigned cluster and a value closer to −1 indicates a bad match (a much better match toward one of the other clusters). A value of 0 indicates that the point could equally well have been assigned to another cluster. The silhouette plot thus provides an alternative view to how well the clustering worked and how much overlap there is between clusters.

Figure 3B strongly confirmed our suspicions that the deterministic clustering would be highly uncertain and should be done probabilistically. Our decision for the number of template maps to derive from the clustering of the EOG and EMG-cleaned data was driven by these two criteria: where the “knee of the curve” was, for the error curve in Figure 3A and looking at the Silhouette plots for different possible values of k. We looked at the Silhouette plots and the temp, and converged on *k* = 5 as that is where the knee of the curve is. Note that we have 4 template maps shown for the cleaned data (in **Figure 6** below). Two of the maps at *k* = 5 proved inversions of each other and as maps are considered regardless of polarity, we combined them (they had *R* = −0.998 for KM-real and *R* = −0.992 for KM-imagined) into one, relabeling all the points of one as belonging to the other cluster. We did this for both KM and FCM, as their behaviors were highly similar. At *k* = 4, they did not converge on the same 4 maps and at higher k, we find cluster centroid-derived template maps get split into multiple maps, including some noisier ones. For clarity, we show only the Silhouette plot for *k* = 6 in Figure 3B. Note that while some of the issues of microstate template derivation can be reduced with the modified K-Means from (Lehmann et al., 1987), the results from that algorithm and the FCM would be less meaningfully comparable. The standard K-Means is able to identify the same underlying microstate template maps, though it may sometimes consider polarity-inverted maps to be two different templates. This can be dealt with after the clustering by combining the two templates and all the points assigned to one can be assigned to the other. This is the approach we took.

### Fuzzy C-means to probabilistically cluster the data

We used the MATLAB Fuzzy Toolbox's implementation of the Fuzzy C-Means (FCM) algorithm (MATLAB, 2017, MathWorks). The objective function minimized is a modification of the K-means objective function, as shown below:

$$\begin{array}{l} J\left(X,\;m,\;k\right)=\overset{N}{\underset{i=1}{\sum }}\overset{k}{\underset{j=1}{\sum }}{U_{ij}}^{m}||x_{i}-c_{j}|{|}^{2} \end{array}$$

Compare this to the standard objective function to be minimized in K-Means:

$$\begin{array}{l} J\left(X,\;k\right)=\;\overset{N}{\underset{i=1}{\sum }}\overset{k}{\underset{j=1}{\sum }}||x_{i}-c_{j}|{|}^{2} \end{array}$$

Note that the FCM objective function adds a membership parameter m, which is a global parameter that specifies the degree of fuzziness of the otherwise linear borders between clusters (just as with standard K-means) and a membership matrix U, which contains (after optimization/training of the algorithm), for each data point to be clustered, the degree of membership to every cluster center c_j_ from the total of k clusters. For *m* = 1, we have standard hard K-means, and for values of *m* > 1, we have progressively fuzzier cluster separations, leading to greater belonging to multiple clusters (and correspondingly smaller values for any given cluster assignment to the given point, especially for nearby and overlapping clusters). The algorithm proceeds exactly analogously to standard KM, with the same initialization (using the kmeans++ selection of the initial centroids), and an iterative improvement of the clusters until a small enough error is reached, at which point the algorithm is considered to have converged. We repeated FCM 10 times (10 replicates) for the final *k* = 5 runs (resulting ultimately in *k* = 4 maps) and run it for a maximum of 1,000 iterations, or until convergence. As with KM, each replicate converged well before reaching the maximum number of iterations, always after at most a few 100 iterations (the numbers of iterations needed increase with increasing k, though stochastically so, due to the randomized initialization of the cluster centroids). The replicate with the lowest resulting objective function value reached during optimization was chosen as the final model to feed into the corresponding MLPs (FCM-imagined and FCM-real), exactly analogously to the KM-based MLP training.

### Softmax multi-layer perceptron probabilistic labeling of microstates

Instead of clustering or running an effectively deterministic decision on the state assigned to any given EEG time point (by picking the template map that has the highest correlation to the given time point), we used probabilistic neural network models (softmax/logistic MLPs) to explore the following questions

*Is the most likely state label assigned with high certainty (*>*95% probability) most of the time? How often is it or is it not? Can we quantify in any new and interesting way the predictability of the EEG microstate assignment to the mean GFP power?*

**Figure 4:**
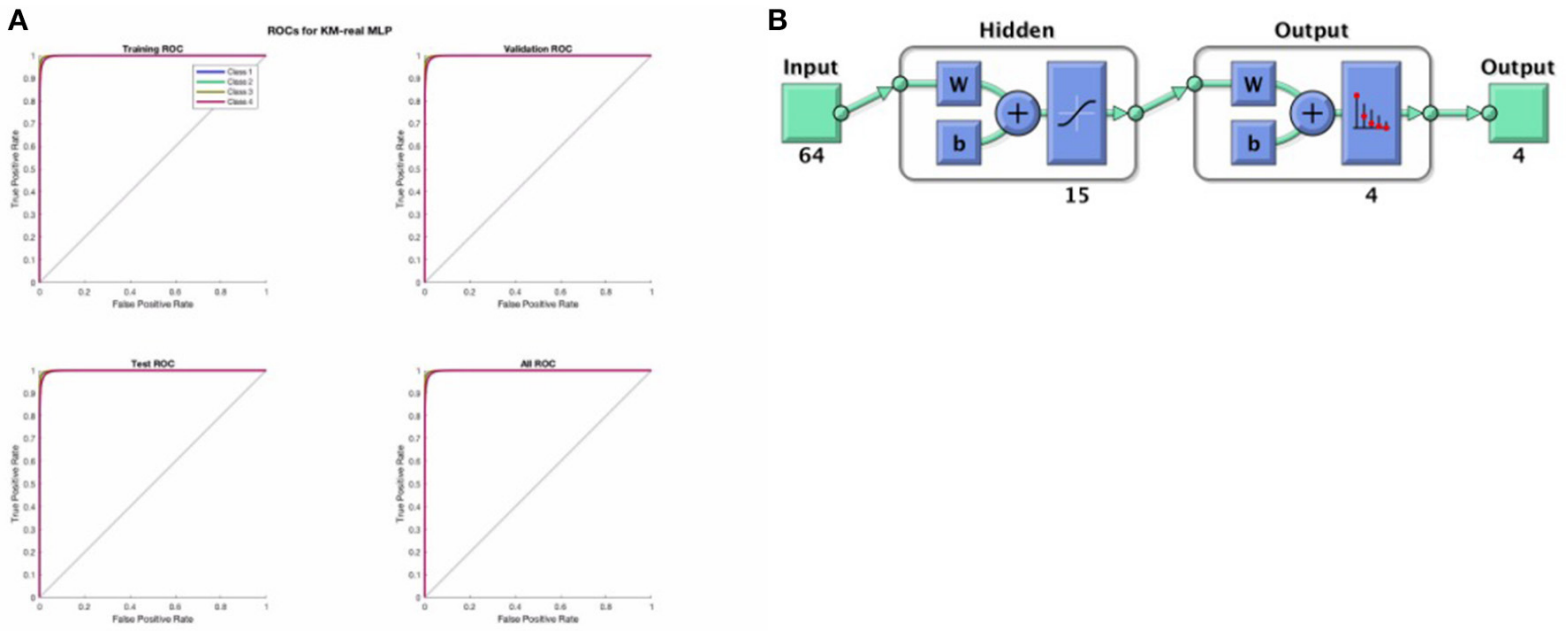
**(A)** Above shows the ROC curves for the training set, validation set and the test set as derived from the input data in a randomized fashion according to a 75-15-15 split, for KM-real. We see that the MLP training was successful and good. **(B)** Shows the architecture of the softmax MLP used for all the MLP models here.

The softmax MLP architecture was a standard feed forward neural network with 1 hidden layer of size 15, and a softmax output layer of width 4 (the 006Eumber of microstates ultimately used) giving probabilistic output where all outputs sum to 1 (i.e., the output function is softmax). One network was trained for each set of clustered images (for FCM-imagined, FCM-real, KM-imagined, and KM-real). Each softmax-MLP was trained using a cross entropy loss function and scaled conjugate gradient descent using a 15-15-70 division of the data accordingly for the test-set, validation-set and training-set, as implemented within the MathWorks MATLAB software's Neural Network Toolbox (MATLAB, 2017, MathWorks). The division is done by picking out data uniformly randomly using the “dividerand” data division function in the Toolbox. Each network was trained for a maximum of 2,000 epochs, or until a stopping criterion (according to a sufficient increase in the validation error after having been previously low) was reached. We re-run the analysis a number of times and all MLP models had finished training sufficiently well before reaching the 2,000 epochs, after a few 100 epochs in fact. We also manually confirmed that each trained network had low training, test and validation errors, as well as a reasonable confusion matrix, before proceeding. We show a typical representative example of the Receiver Operator Characteristic (ROC) curves indicating the success in the training, validation and test sets of KM-real, shown in Figure 4 below.

Figure 4 above shows a representative ROC curve for the cleaned data MLPs. All trained MLPs had highly similar successful ROC curves however, also in the non-EOG and EMG cleaned data.

Finally, note that the hidden units used standard optimal tanh nonlinearity and the output units had values that sum to 1, performing a softmax classification.

### Template map matching and re-labeling

Because both KM and FCM are randomized models, and the cluster/template labels are assigned randomly, the resulting templates, though they may be corresponding/matching, have different nominal labels. To simplify analysis and comparison between models, we correlated all templates between models and sorted the maps according to their correlations. This gave us a mapping between the generated templates from any two models. We re-labeled all the training data for the MLPs according to the template labels of the FCM-imagined labels. This choice was arbitrary and any of the four model templates could have been chosen as the one to label against. Since the map correlations were very high, either identical with *R* = 1 or very close to that, the model output to re-label against was not important. This step was important so that the subsequent MLP outputs for all 4 models (FCM-imagined, FCM-real, KM-imagined, KM-real) all had comparable outputs, with the same label for the same microstate.

### General linear models (GLMs) to look at relationships between the GFP and MLE probability

Finally, we fed the GFP and the mean probability of the MLE label from the MLPs into general linear models (GLMs), one for each data and model condition (i.e., FCM-imagined, FCM-real, KM-imagined, KM-real), to model any relationships between the prediction probability of the most likely label assigned by the MLP for each model and the corresponding data's mean GFP.

## Results

### Template map similarities and differences between real and imagined motor movements between FCM and KM

**Figure 5:**
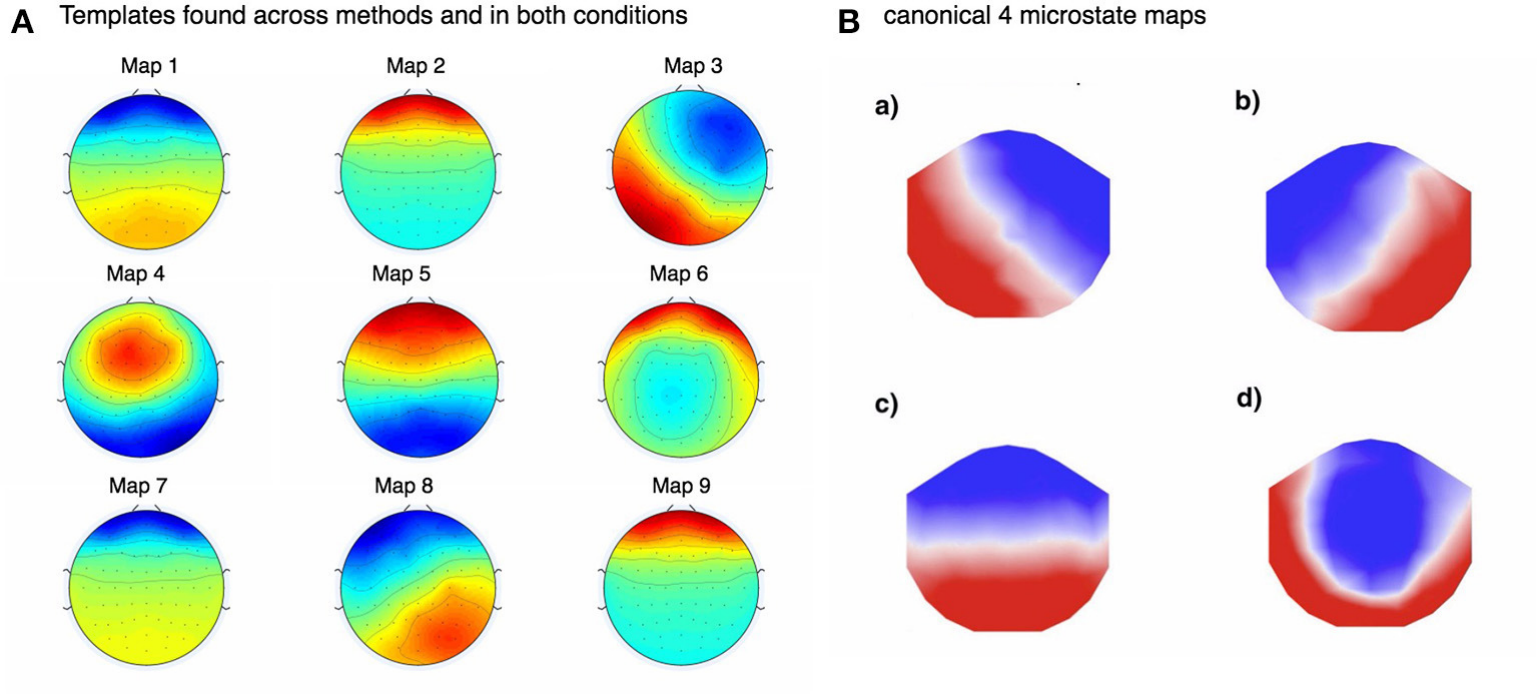
**(A)** Above shows the template maps found using FCM for imagined motor movements in pre-EOG and EMG cleaned data. Due to the very high correlations between the templates found in imagined and real movement data, as well as between FCM and KM (see Figure 6), we only show these templates. **(B)** was adapted from (Britz et al., 2010). Note that some of the found template maps are almost identical to the typical (what we call here “canonical”) maps found by others. For example, our template 3 maps to A in 5b, 5 to C from 5b, 8 maps to B in 5b. Template map 4 may map to canonical template D. This shows that, perhaps somewhat surprisingly, lack of EOG and EMG cleaning does not prevent the convergence to the underlying microstate template maps.

**Figure 6:**
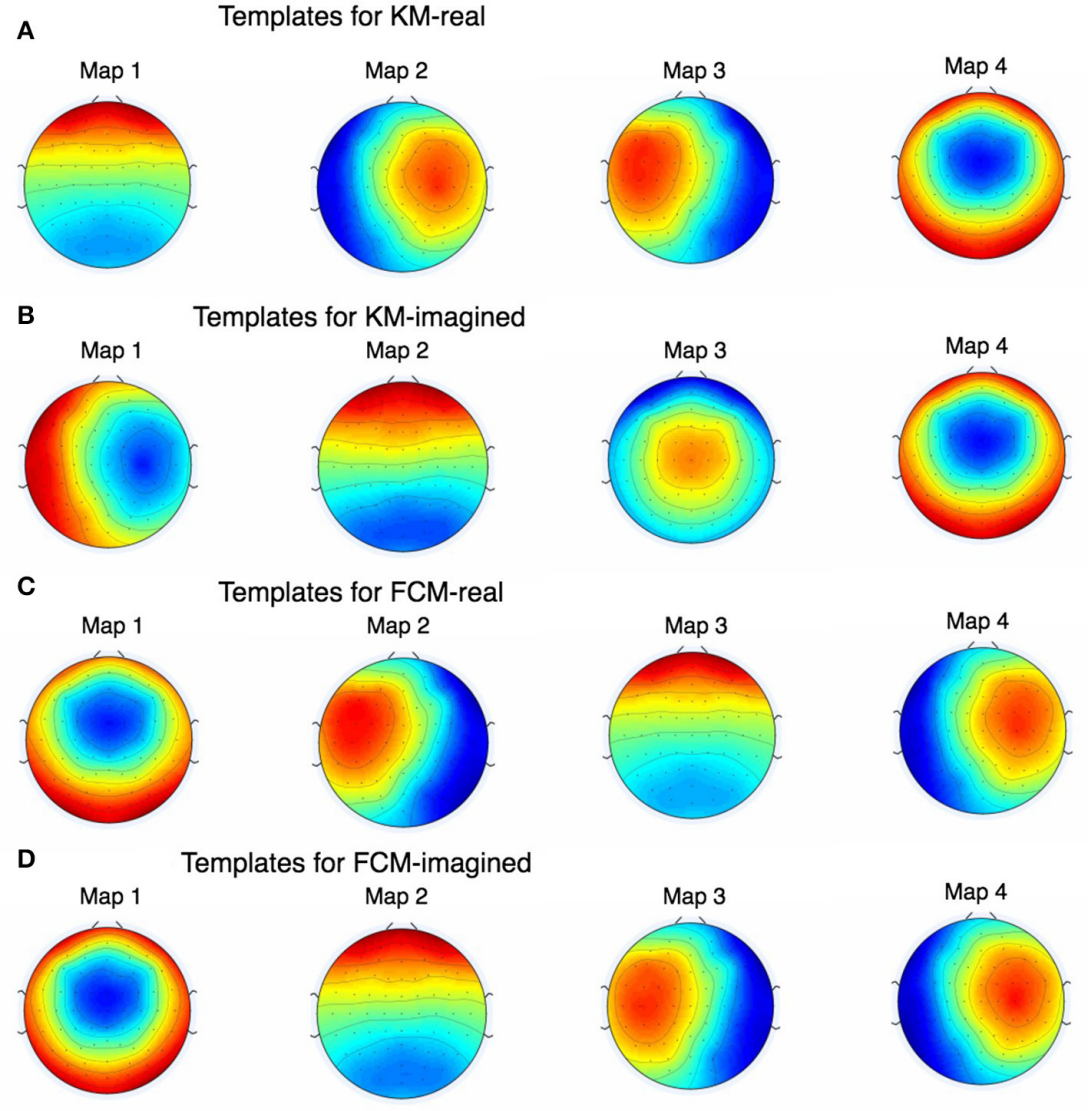
The figure shows all the EOG and EMG-cleaned maps as found by KM and FCM. Note that the maps are highly similar but KM-imagined in **(B)** did not converge (even after multiple repetitions) on quite the same map as KM-real in **(A)** for one of them. During development of this work and prior to the results we report here, we found this behavior of K-Means to occur often and rarely, if ever, for FCM, which is shown in **(C,D)**.

**Figure 7:**
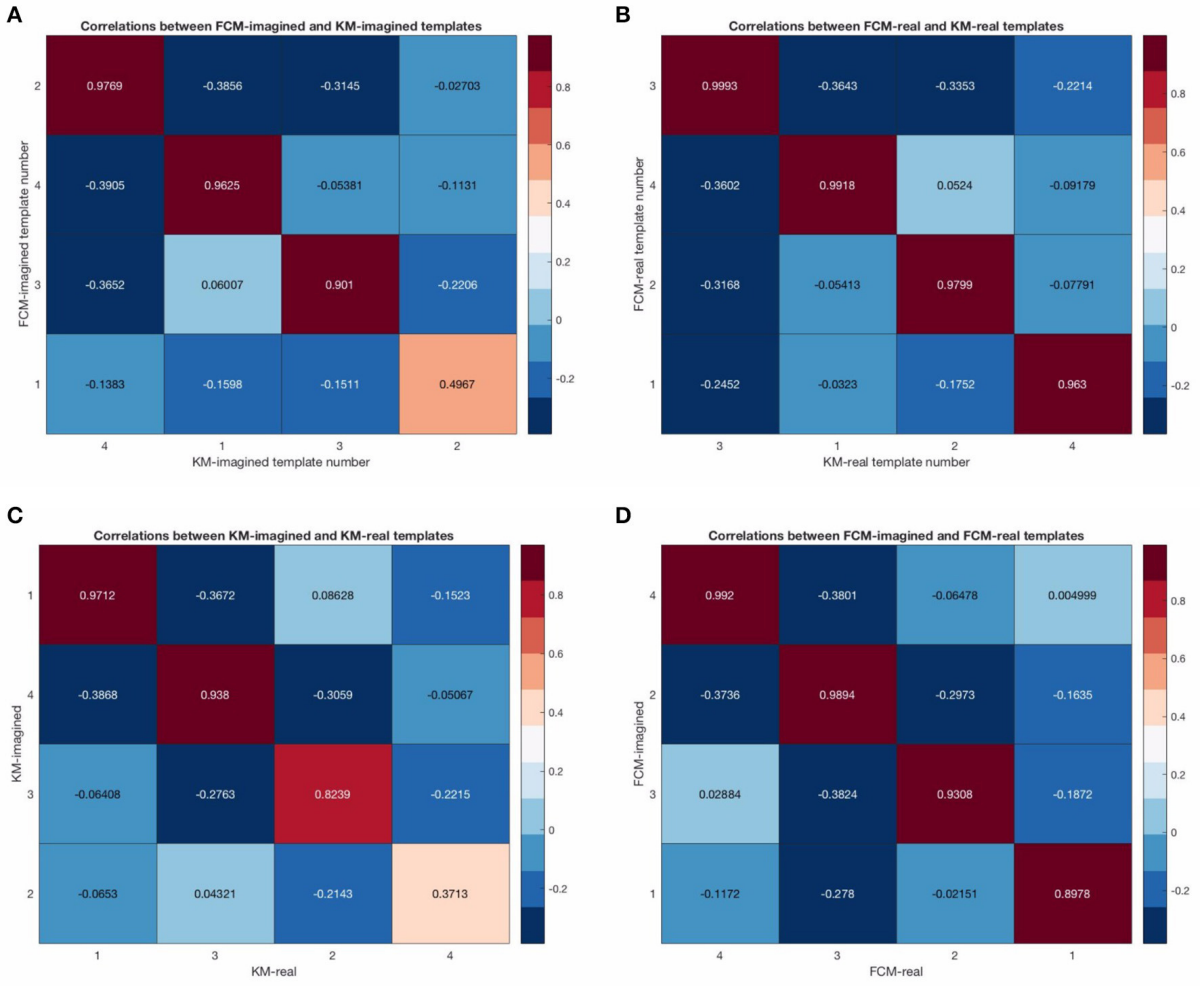
Correlation matrices between the cleaned template maps. Sub-figure **(A)** shows the template correlations between FCM-imagined and KM-imagined. **(B)** shows the template correlations between FCM-real and KM-real. **(C)** shows the template correlations between KM-imagined and KM-real. **(D)** shows the correlations between FCM-imagined and FCM-real. Note that the single lowest template correlation is due to the map 3 from KM-imagined.

In Figure 5 we show pre-cleaning derived template maps. Note that though the maps differ from post-cleaning template maps (shown in the next Figure 6), the non-noise maps are nevertheless successfully found. This was the case for both KM and FCM, as the correlations between the pre-cleaning derived template maps is mostly very high between the 4 models—i.e., between the FCM models, KM models and between the corresponding FCM and KM models for real and imagined (see Figure 7). The notable exception is map 3 in KM-imagined, which does not correlate well to most of the maps in KM-real or the FCM-derived maps. However, some relationships and template maps did not stand out as strongly without EOG and EMG-cleaning (see Figure 5, where multiple noise maps were also found). Perhaps somewhat surprisingly though, most of the same templates were found, as shown in Figure 5, without any EOG or EMG artifact cleaning on the data.

Figure 6 shows the final 4 template maps found after EOG and EMG cleaning of the data. The maps are re-arranged according to maximal similarity to the canonical microstates A, B, C, and D sequence. Note that in both pre- and post-cleaning, we found the three of the templates often found and reported in the literature (shown in Figure 5B) that we call here the “canonical” templates, in particular because they are found using canonical (i.e., currently and typically used deterministic) clustering approaches. This is in particular the case for the first, second and fourth maps for KM-real, KM-imagined, FCM-real and FCM-imagined. Note that the labels on the Figure 6 correspond to the different labels assigned by each of the multiple runs of the KM and FCM. However, map 3 in KM-imagined (which is presented as the 2nd map in the sequence) is less clearly relatable to any of the 4 canonical maps. It is perhaps similar to and identifiable as map 10 in Yuan et al. (2012). We suggest an explanation for this lack of correspondence of KM-imagined to the canonical template B (bottom right to top left diagonal scalp distribution) in the discussion.

### Probabilistic analysis from Fuzzy C-means and the softmax MLPs

**Figure 8:**
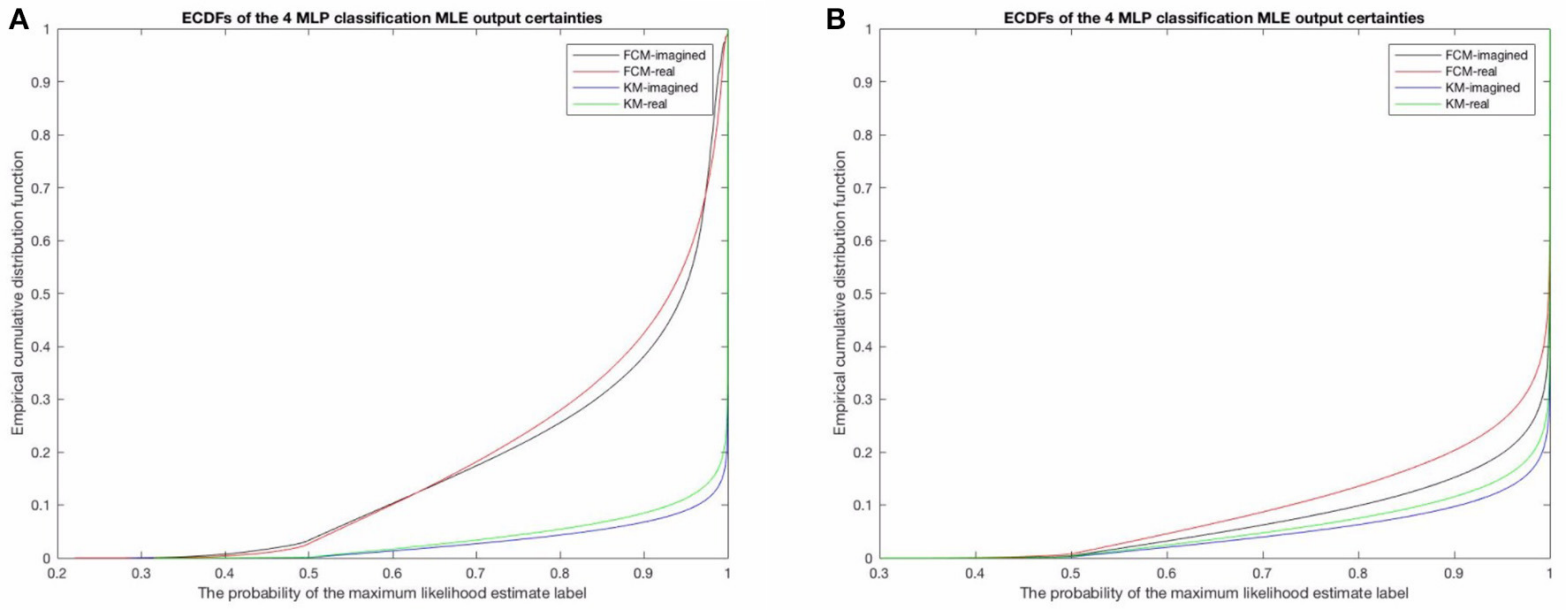
**(A)** Here shows the probability of the maximum likelihood estimate (MLE) as assigned by the MLPs in the uncleaned data and **(B)** shows the results for the cleaned data. Note the difference between the FCM-derived probabilities of the microstate sequences for both real and imagined movements, compared to the KM-derived sequences, in both the uncleaned/raw EEG to the left and the cleaned EEG on the right. This is likely a result of the KM not taking into account similarities and overlap between microstate templates and the specific locations of the EEG time series where these overlaps should be taken into account. Noteworthy is also that both methods do however find a very similar difference between imagined motor movements (black line and the blue line) and real motor movements (red and green lines). The real motor movements are somewhat more predictable in both the raw EEG and the cleaned EEG.

We investigated the differences in the probabilistic MLP outputs between the probabilistic FCM-derived and deterministic KM-derived sequences. In particular, the empirical cumulative distribution function (ECDF) between the two shows that there is considerably higher variability in the assigned probability to the MLE label for any given time point (see Figure 8A below for this result for the raw and uncleaned EEG and Figure 8B for the cleaned EEG) in the FCM models than for the KM models (obviously, due to the non-probabilistic input to the KM MLPs). The relationship between imagined motor movements and real motor movements shows a similar difference for both FCM and KM—namely, that imagined motor movements tend to show a somewhat lower predictability (lower probability of the MLE state assigned). This effect is shown to be significant in the FCM-derived sequence, as over 40% of the points have a lower than 90% probability assigned to them in the artifact-contaminated data. For both imagined and real motor data, FCM reveals that up to 10% of the points are assigned a probability of only 60% to the most likely state (MLE state). Typically, the rest of the probabilities (in order to sum to 1 for any given prediction) tend to fall into only one or at most two of the other states, and so a probability of 50–60% implies that in those times, the second most likely template may in fact be the correct template to assign to the given time point. Cleaning of EOG and EMG did result in more certain predictions, though with significant uncertainties remaining in both KM and FCM-derived sequences. Again, note that imagined movement data lead to higher MLP prediction uncertainties for both KM and FCM-derived microstate sequences. This helps affirm that the results are not purely algorithm-dependent. In both cleaned and uncleaned data, we find that FCM suggests higher uncertainties in the final assigned microstates. We discuss this the potential implications of this in the discussion below.

**Figure 9:**
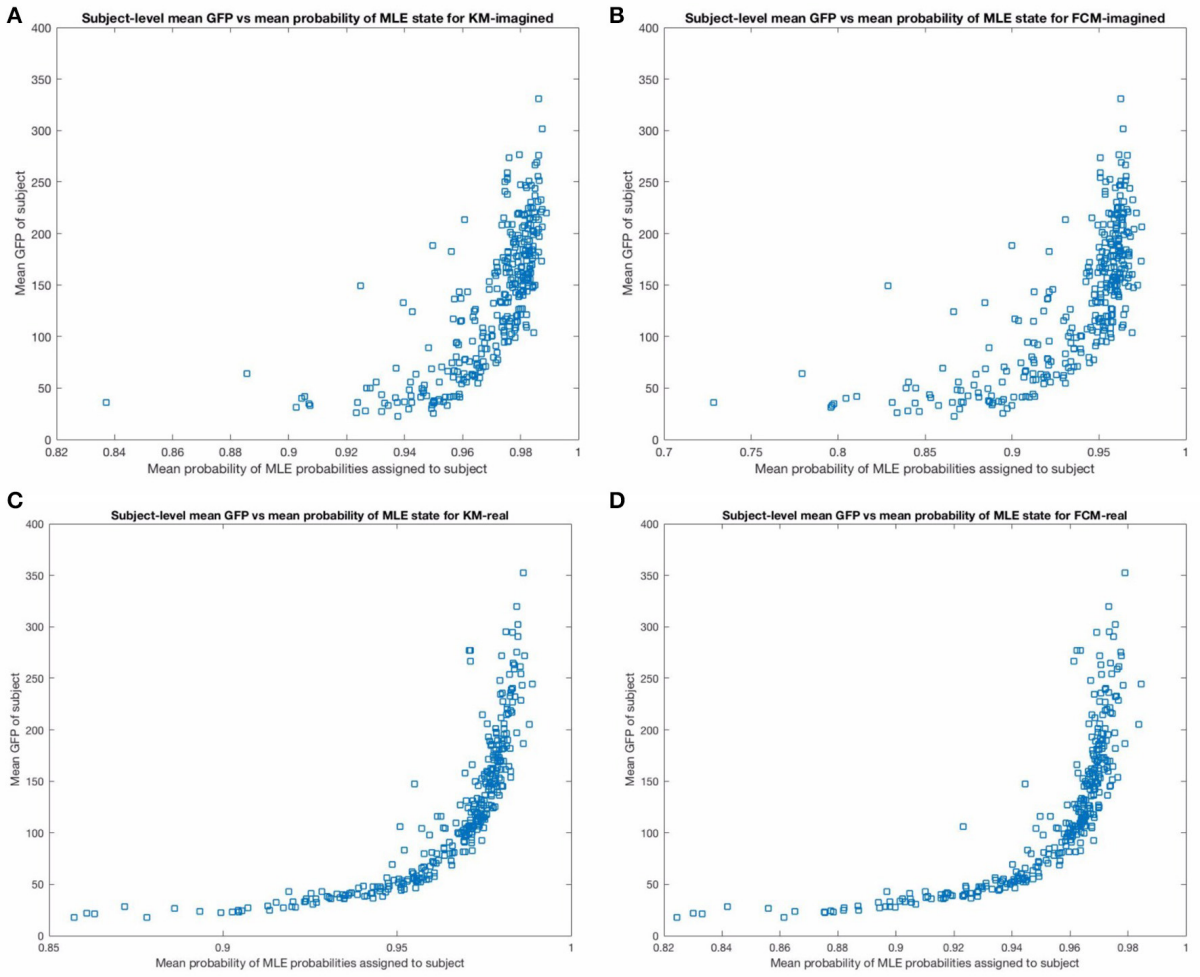
The figure above shows the mean GFP (on the y-axis) vs. the mean probability of the maximum likelihood estimate state from the corresponding MLP (on the x-axis), across subjects. The top **(A,B)** show these for imagined motor movements and the bottom **(C,D)** show these for real motor movements. Note that the relationship is less noisy and stronger for the real motor movements for both FCM and KM. In particular, there are more subjects with significantly lower average certainty in the microstate assigned in the imagined models than in the real motor movement models. We excluded one outlier point from the top two sub-figures in the imagined motor movement data that had a mean GFP of close to 800, for a clearer comparison between the graphs.

We then performed a subject-level analysis, exactly analogously to the group-level results presented above, investigating the relationship between the probabilities and the GFP. As expected, there are strong relationships between the GFP and the uncertainties in the microstates found—indeed, the relationship was more pronounced than we expected (see Figure 9 below). This confirmed part of our initial supposition that the GFP will have a strong effect on the predictability and certainty in the microstate prediction. We also find (unsurprisingly) that the variance in the MLE predictions on a subject-level correlate very strongly with the mean GFP for the respective subject. When plotted (not shown for brevity) we find that the prediction certainty varied least for subjects with the highest mean GFP levels (compare to Figure 9).

## Discussion

Both the probabilistic FCM and the deterministic KM-derived microstate sequences found highly similar templates, and subsequent prediction probabilities as inferred by the respective MLPs. We found all 4 of the “canonical maps” shown in Figure 5B, which have been shown in a combined EEG-fMRI study by Britz et al. (2010) to be highly correlated to certain cortical areas, as revealed by BOLD fMRI imaging. In particular, map A taken from that study was found to negatively correlate to BOLD signals from areas involved in phonological processing, and partly positively correlate to primary visual areas. Map B was found to be negatively correlated to non-primary (extra-striate) visual areas. Map C was found to correlate positively with BOLD activation in fronto-insular areas involved in body representation and map D was negatively correlated with BOLD activation in fronto-parietal networks involved in attentional re-orienting and switching. We did not significantly investigate the dynamics of these found microstates as the focus of this study was primarily on predictability of microstates using new probabilistic methods. The few microstate switching dynamics that we did look into suggested some small differences between imagined and real motor movement microstate transition probabilities (not reported) in FCM-derived sequences but no significant differences in the KM-derived sequences. We plan to investigate the microstate dynamics using probabilistic methods in detail in subsequent studies. We may restrict analysis according to the mean GFP of the subject, or only focus on regions of the EEG with high enough GFP, as Figure 9 indicates a very strong relationship between the mean GFP of the subject and the certainty in the predictability for both KM and FCM and for both imagined and real motor movements. The effect is weaker for imagined motor movements but still strong (see the top 2 sub-figures in Figure 9). While we were surprised by the strength of the effect, this does confirm the literature's long-standing focus now on deriving microstates only at GFP maxima. However, our results here strongly suggest to us that our original hypothesis about the actual GFP strength, as opposed to the fact that we are looking at a GFP peak, is what influences the SNR. We suggest that the current approach of deriving microstates at GFP peaks is in fact introducing noise as it is fully based on the local properties of the signal. What we believe current and future researchers should do is focus on all EEG time points that are above some threshold level of GFP, though a careful and thorough analysis of the relative merits of a local peak-detection based approach (as currently done in the literature) and a global mean GFP threshold-based approach should probably be done to confirm empirically that this is indeed the case.

Performing probabilistic analysis on EEG data shows us that it does not seem to perform any worse than deterministic clustering and may in fact be somewhat better—e.g., see the template maps found in Figure 6. We also find that FCM suggests a higher uncertainty in microstate assignment than KM, which may be due to the probabilistic nature of the method as it distributes the certainty of microstate label assignment at least to some degree, or it could also be in part that some microstate assignments are inherently uncertain and these uncertainties are being picked up better. Note for example in Figure 3B that the Silhouette plot strongly suggests that the cluster assignments, despite being “optimal” (in the sense that K-Means seemed to have converged to the global optimum there), are highly uncertain. This was already strongly suggestive that the deterministic KM-driven clustering is far from “perfect.” FCM Silhouette plots (not shown) show very similar Silhouette plots, which is unsurprising, as the FCM algorithm works very similarly to KM and the maps we converged to are overall highly similar. It would be worthwhile to compare how the modified KM algorithm typically used in the literature would fare here, as well as an equivalently modified FCM clustering approach. We hope to report back in subsequent work on how a modified FCM performs compared to the analogously modified KM algorithm.

We confirmed in another way that the variability in imagined motor movement microstates is noticeably higher than in real movement microstates. We used SOMs during the early stages of this study to try to find on some other approach for empirically determining the optimal number of clusters/templates. SOMs can be argued to be an alternative method for microstate analysis in that the technique was designed to re-represent high dimensions data (in this case 64 dimensional) into 2 dimensions, which is highly appropriate for the problem situation here. This is particularly relevant to EEG data n that the channel data, in particular when looked at topographically, as it is in microstate analysis, is quasi-2D. We suggest therefore that the SOM approach to clustering and deriving microstate template maps is likely to prove fruitful and should be explored more thoroughly. There are also interesting variants of SOMs, such as growing neural gas or growing when required network (GWRN), which could both be powerful additional alternative approaches to be tried in future uses—as they have the ability to select the required number of neurons/clusters in different ways than the traditional cross-validation approach employed in the literature.

It is promising that the MLP predictability results correlated so well with the mean GFP on a subject-level. This served as a very strong “sanity check” that the neural-network driven method is working well (though also the training, including some amount of generalization ability, proceeded very successfully, as Figure 3A indicates). Figure 9 shows that the prediction error asymptotes at mean MLE probability of 1 (of course). This asymptote that is neared but not quite reached at the highest mean GFP level subjects suggests that all microstate assignments are bound to be somewhat uncertain regardless of the method employed. This approach to quantifying the relationship on a subject-level between the GFP and the predictability of the current microstate is, to the best of our knowledge, novel. For one, it allows not only for a precise and fast assignment of microstates but quantifying how certain we are that the brain is in a given microstate at any given time point. In such cases where any uncertainty exists (which is always the case, more or less), that uncertainty should, in general, be quantified using some type of probabilistic technique, as we have done. Interestingly, this relationship is more variable for imagined motor movements compared to real motor movements and, to a much lesser extent, for FCM than for KM-derived sequences. We suggest that this higher uncertainty in imagined motor movements is likely to be due to the more exploratory nature of the cognitive state during imagining of motor movements compared to the performing of real motor movements.

Note that we did not make full use here of the probabilistic nature of the FCM, as we used only the most likely (maximum likelihood) labels derived from the FCM in training the corresponding FCM MLPs. However, that in part defeats the purpose of doing probabilistic clustering, as we are throwing away significant information by focusing only on the most likely template. There are a number of ways that the full probabilistic vector of assignments from FCM can be fed to the FCM MLPs in order to make use of them—e.g., we can train non-softmax regression MLPs using Mean Squared Error (MSE) instead of cross entropy. While we have begun to explore this approach, it is less directly comparable to the KM-driven MLP training than what we have done here. However, it is also likely to be more revealing of the underlying microstate dynamics by making fuller use of the microstate uncertainties. Another interesting venue to explore in delineating the variability in microstate assignments would be to follow the modified K-Means template cluster derivation by some probabilistic assignment of microstates to the EEG time course—e.g., by running the resulting vector of topographic correlations or the vector of distance measure, derived using any of a number of non-linear distance measures (e.g., using Dynamic Time Warping) through a softmax, to derive probabilistic belonging to microstates. These probabilistic vectors of template similarity to the current EEG time point can then be fed, as described above, to train a multi-output neural network to do regression with the chosen or found templates as outputs and the belonging to each of them as the target values. The use of neural networks for assigning microstate labels as used here is, to the best of our knowledge, novel. It opens a number of new avenues of potential research. For example, we used a single hidden layer softmax MLP in each case, but we are now investigating the use of deeper networks in future work. Multiple layers may reveal underlying hierarchical relationships, which deep neural networks are very good at finding, especially for low-order polynomial hierarchical relationships. Such relationships might reveal novel similarities and differences between the templates and the underlying large-scale functional networks. Also, we chose to model real and imagined motor movements in separate neural networks, but there are a number of combinations that we could have tried as well. For instance, we could have modeled FCM and KM-derived sequences together using the same MLP and investigated the ability of one to model the peculiarities of the other method. We could have done the same for real and imagined movements as well. The latter could potentially suggest new correlations, especially when combined with a deeper network, between multiple cognitive states and how these correlations interact. We also did not look into the different MLP dynamics in time—is the predictability lower or higher earlier or later on in a task? Finally, we did not explore how probable the most likely next microstate label is given the current microstate label. Or: how many future steps can we predict with somewhat certainty (e.g., probability > 0.5) given the current microstate label and its certainty. Because EEG and EEG microstates are both known to exhibit long-range correlations and 1/f dynamics (Shew et al., 2009; Fagerholm et al., 2015), reflecting the underlying large-scale BOLD-imaged brain networks that are now thought to be the same phenomenon, we expect that despite the highly variable and non-stationary nature of the EEG signal, we would find diminishing but non-trivial predictive ability many time steps in the future. The use of probabilistic MLPs gives us a powerful and flexible method for exploring these questions. We are now investigating some of these issues, but wanted to share some of our methodological results. The data set used is large and of high quality, and this is helpful in training deeper neural networks or other models with many parameters and we recommend use of this data set. By performing multiple studies on the same data set, we can also more easily compare results with others. We suggest that this one would be a valuable reference data set, given the high number of relatively clean data points and as such will likely be using it for future microstate research ourselves. Furthermore, we decided on presenting both uncleaned/raw EEG results and the results from the cleaned/preprocessed data. The reason for this was to suggest that cleaning may not be a necessary step for microstate analysis if the right methods are used, which can disentangle and assign template maps well enough regardless. While we did not explore this point in detail, we show that most of the dominant scalp topographies found, shown in Figure 6, are also found in Figure 5 from the uncleaned data. Perhaps similarly to how deep neural networks allow for automated feature extraction without preprocessing and feature engineering, neural networks may lead to a more automated microstate analysis framework. Additionally, one particular interest of ours is that of attempting to compute microstates from a very small number of channels (e.g., a few, for example 5, as found in some cheap and now widely-available commercial EEG headsets). Such an approach would be both informative and practically useful toward real-world applications. This would significantly increase the real-world potential application of microstates. Deep and other neural network architectures may help in this quest by virtue of its powerful predictive and flexible abilities. While (Khanna et al., 2014) showed that group-level microstates are reliably identifiable with as few as 8 electrodes, the variability of the microstate templates was far higher when the maps were computed on a subject-level. As we propose in this paper, it is also not clear that “reliability” of the maps found with standard methods is We are now also exploring the use of this MLP-based approach by using dense EEG (e.g., 64 channels as in here) but attempting to model and predict microstate dynamics with only a small subset of the channels and comparing the performance and reliability between doing this with the raw EEG and with cleaned EEG, using softmax deep neural networks.

One potential translational use of microstates in conjunction with quasi-automatable real-time closed-loop learning machine approaches such as reinforcement learning (RL). A RL algorithm's current state can be defined based on the current microstate, providing a somewhat directly interpretable definition of the states used in the RL system. Since RL and microstate classification (especially with pre-trained neural networks) can both run efficiently and in real-time, this suggests a use of EEG microstates for more interpretable neurofeedback BCI or BMI applications (in terms of being representative of large-scale functional network activity as observed also through fMRI, while having obvious advantages to not being fMRI). If decisions are to be made based on the current global brain state (e.g., for neurofeedback or BCI), it would be advantageous if they were made probabilistically, rather than deterministically, given that there is considerable uncertainty in microstate classification and dynamics—and at least some of this uncertainty may be informative of the underlying behavioral and neural dynamics, as suggested by some of the differences between imagined and real motor movements found here. In the present work, we found rare but existent occasions when the certainty in the most probable label is around or even below 0.5. If critical decisions are to be made based on brain state, they should not be made carelessly in a low certainty state and the probability of the current microstate label being correct should be taken into account. This is especially relevant for BCI applications—and most notably for strongly mission or life-critical real-world use cases.

Due to some of the notable differences in the microstate definition between probabilistic FCM vs. deterministic KM, we suggest that future microstate studies at least use multiple clustering methods in parallel, to compare how the microstate behaviors are affected. “Small” differences in clustering algorithms can potentially have cumulatively significant effects in real-world situations. Using probabilistic methods to both cluster and assign microstates should lead to greater elucidation of the complex relationships between microstates and other measures of brain dynamics (including, but not limited to, BOLD, EEG cascades, mean GFP or, roughly speaking, baseline EEG power variability, etc.). While many methods will produce similar microstate sequences, superficially, there may be “hidden” patterns in the data that can only be shown using one method (here Fuzzy C-means) as opposed to another (e.g., K-means). FCM is a natural extension and modification of KM and the algorithm proceeds very similarly to the K-Means algorithm, though it has a somewhat higher running time and complexity than KM. An interesting alternative that we explored the use of was Gaussian Mixture Models, but it involves fitting more parameters and we could not get this approach to perform sufficiently well to be compared against KM. One approach to initialize the probabilistic GMMs is by using the KM or FCM-derived centroids and variability inferred from them as the means and variance-covariance matrices for the GMM initialization. This is what (Lucia et al., 2007) did in deriving topographic views of event-related potentials from individual trials. Unfortunately, they assigned microstates using the maximum posterior probability—i.e., the most likely component of the trained GMM, thereby discarding most of the probabilistic model's usefulness. We did not pursue GMM use in detail as the technique is less directly comparable to KM. Nevertheless, such a probabilistic approach is a step in the right direction. While our FCM-driven approach has the same issue as their GMM maximum posterior probability template assignment, one “redeeming” feature of our work here is that the subsequent assignment and analysis is fully probabilistic.

To summarize, one possible picture that has emerged from this probabilistic approach is that imagining motor movements involves blurrier microstates and transitions between these. We suggest that this is a general feature of cognitive states closer to the exploratory resting state-like modes of brain dynamics, as it is well known that, in general, resting state dynamics involve faster changes and are closer to critical dynamics than more focused cognitive states. Further careful microstate studies will clarify the “hidden dynamics” of microstates and their relationship to other neural phenomena and probabilistic derivations of microstate templates as well as probabilistic assigning and modeling microstate dynamics is likely to help in this. While microstates have historically always been assigned in a mutually exclusive fashion—i.e., that a given EEG time point is either in one microstate or another—this is obviously a gross oversimplification. While we do not suggest that a global dominant brain state does not exist, as suggested by all the previous microstate research, quantifying the uncertainties involved in this binary assignment decision is surely going to prove informative in better delineating the underlying brain microstate dynamics. We therefore finish by highlighting the importance of using neural networks as their flexibility and computational simplicity, especially post-training, will allow for new real-time and real-world uses of EEG microstates. It is also conceivable that the use of deeper neural networks may find hidden hierarchical dynamics of the microstates. We leave this last and other issues we have raised to be examined in future work by ourselves and others.

## Online code repository

All the relevant code used to derive the results in this paper is available on github at: https://github.com/martindinov/probabilistic_microstates/.

## Author contributions

MD and RL both designed and contributed to the research, MD wrote code and analyzed data, MD and RL both wrote the paper.

### Conflict of interest statement

The authors declare that the research was conducted in the absence of any commercial or financial relationships that could be construed as a potential conflict of interest.
